# Quantitative Analysis of Temporomandibular Joint Adaptations After One Year of Complete Denture Use: A Cone-Beam Computed Tomography Study

**DOI:** 10.7759/cureus.76662

**Published:** 2024-12-31

**Authors:** Pradeep Dilip Taide, Auroosa H Mirza, Premraj Jadhav, Medha Upadhyay, Natasha Bathla, Prerika Agarwal

**Affiliations:** 1 Department of Prosthodontics and Crown and Bridge, Yogita Dental College and Hospital, Khed, IND; 2 Department of Prosthodontics and Crown and Bridge, Kothiwal Dental College and Research Centre, Moradabad, IND; 3 Department of Prosthodontics and Crown and Bridge, Luxmi Bai Institute of Dental Sciences and Hospital, Patiala, IND

**Keywords:** complete denture, condylar, cone beam computed tomography, edentulous, temporomandibular joint

## Abstract

Introduction: Alterations in occlusal relationships in individuals with complete edentulism considerably disrupt the equilibrium of the stomatognathic system. Evaluation of the temporomandibular joint (TMJ) is crucial during the edentulous phase, as it influences both aesthetic and functional outcomes. This investigation sought to assess alterations in condylar positioning one year after the placement of complete dentures in edentulous patients, with additional objectives to examine variations based on sex and side.

Materials and methods: This cross-sectional prospective cohort investigation was carried out in the Department of Prosthodontics, involving 15 participants who were systemically healthy, aged between 45 and 60 years, completely edentulous for a minimum duration of one year, and were first-time denture recipients. Following denture insertion, a one-year follow-up evaluation of condylar positioning was conducted using cone-beam computed tomography (CBCT). Analyses were performed on the intercondylar angle, interocclusal gap, and TMJ space (anterior, posterior, superior, and medial). Statistical evaluations were performed using paired and independent t-tests. The significance threshold was set at P < 0.05.

Results: The mean ages of the male and female participants were 55.43 years and 56.38 years, respectively. Significant changes were observed in the TMJ spaces and condylar dynamics at the one-year follow-up. The posterior, superior, and medial joint spaces decreased significantly (p = 0.001), indicating the posterior, superior, and medial displacement of the condyle. The interocclusal gap increased significantly from 1.47 mm in males and 1.51 mm in females at denture insertion to 1.79 mm and 1.80 mm, respectively, at follow-up (p = 0.001). The intercondylar angle significantly decreased (p = 0.001). Sex-based differences were noted in the medial joint space and condylar angle on the left side, with larger mean changes in males (p < 0.05). Side-based differences revealed greater superior space, medial space, and condylar angle on the right side than on the left (p < 0.05).

Conclusion: This investigation revealed substantial morphometric alterations in the TMJ spaces and condylar positioning following one-year post-complete denture placement, characterized by pronounced increases in interocclusal gap and reductions in intercondylar angle. Consistent monitoring and prompt repair or replacement of dentures are essential for preserving the TMJ integrity and occlusal equilibrium in edentulous individuals.

## Introduction

Disruption of the occlusal relationship in individuals with complete edentulism can significantly impair the equilibrium of the stomatognathic system [1]. Assessment of the temporomandibular joint (TMJ) is crucial for the appropriate application of dentures during the edentulous phase because it contributes to both aesthetic considerations and nutritional practices [2]. Furthermore, this evaluation has played a pivotal role in enhancing awareness regarding the necessity of denture use during the edentulous period, given that the continuation of edentulism may lead to specific mandibular dysfunctions or disorders, ultimately affecting the overall health of patients [3].

Based on statistical evidence, the geriatric demographics of developing nations have experienced a significant increase over the last few decades [4], accompanied by various modifications that seem to illustrate an adaptation of the condyle, articular disc, and articular eminence [1]. Prolonged complete edentulism may result in multiple irreversible deformities such as anatomical alterations of the TMJ. Meticulously crafted full dentures seek to restore occlusal equilibrium and enhance the stability of the TMJ, thereby facilitating the restoration of condylar movements within a more physiologically acceptable spectrum [1].

A study by Singh et al. compared TMJ changes by cone-beam computed tomography (CBCT) in pre- and post-complete denture patients, and it was concluded that a correlation existed between the flattening of the articular eminence and advancing age; conversely, the extent of deformation was observed to be significantly higher in individuals who were completely edentulous when compared to those maintaining a normal occlusal relationship [5]. However, they did not evaluate changes in the condylar position bilaterally in these patients or changes in the TMJ at follow-up visits. Therefore, the present study was conducted with the primary aim of evaluating changes in TMJ spaces one year after complete denture usage in edentulous patients. The secondary objective was to assess sex-based and side-based differences in TMJ spaces in such patients.

## Materials and methods

Study design and setting

This cross-sectional prospective cohort study was conducted in the Department of Prosthodontics, Kothiwal Dental College and Research Centre, Moradabad, India, between January 2022 and May 2024 to evaluate the changes in TMJ spaces and condylar position in completely edentulous patients following the insertion of conventional complete dentures. Ethical approval (KDCRC/IERB/12/2021/56) was obtained from the institutional ethics review board and written informed consent was obtained from all participants.

Sample size estimation

Sample size analysis was performed using the G*Power software, version 3.6.9 (Heinrich-Heine-Universität Düsseldorf, Düsseldorf, Germany). Fifteen samples were determined to be sufficient for the present study. The estimated sample size was found to be 12 patients, calculated with 80% statistical power and a 5% alpha error, considering a minimum effect size of 0.67 for the intercondylar angle in edentulous patients. Considering the loss to follow-up, the sample size was increased to 15 patients.

Study population

Fifteen participants aged 45-60 years were selected based on the following inclusion criteria: systemically healthy patients who were completely edentulous for at least one year and were first-time denture wearers with Angle’s Class I ridge relationship, with no prior history of TMJ disorders, absence of any radiotherapy, absence of any systemic conditions affecting the TMJ, and willingness to participate in follow-up assessments. Exclusion criteria included poor neuromuscular control, congenital or acquired craniofacial abnormalities, poorly resorbed ridges, previous prosthetic rehabilitation, or conditions affecting mandibular movements.

Methodology

The assessments were performed using CBCT to evaluate the TMJ spaces and condylar positions in centric relation. Additionally, clinical examinations were conducted to assess the interocclusal gap or freeway space, which is defined as the distance between the upper and lower jaws when the mandible is in a physiological resting position. Complete dentures were fabricated for all participants following conventional prosthodontic protocols, including accurate impressions, jaw-relation records, and occlusal adjustments to ensure proper fit and function. Hanau articulator (Whip Mix, Farmington Avenue, Louisville, Kentucky) was used. After insertion, participants were instructed on the use and maintenance of their dentures. Subsequent to fabrication, a laboratory remounting process accompanied by selective grinding was implemented to rectify any processing inaccuracies and preserve the attained balanced articulation (BA). Dynamic bite registration was performed in all patients using a Gothic Arch Tracer (Massad Intraoral Establisher®, Tulsa, Oklahoma). The dentures underwent clinical verification to ensure simultaneous bilateral occlusal contact, devoid of any gliding in the centric relation, and free from interference during excursive movements. Clinical remounts and occlusal adjustments were performed to enhance occlusal equilibrium prior to the final placement. All the patients were recalled for follow-up visits (initially after two days, then after one week, and then every three months till one year). 

**Figure 1:**
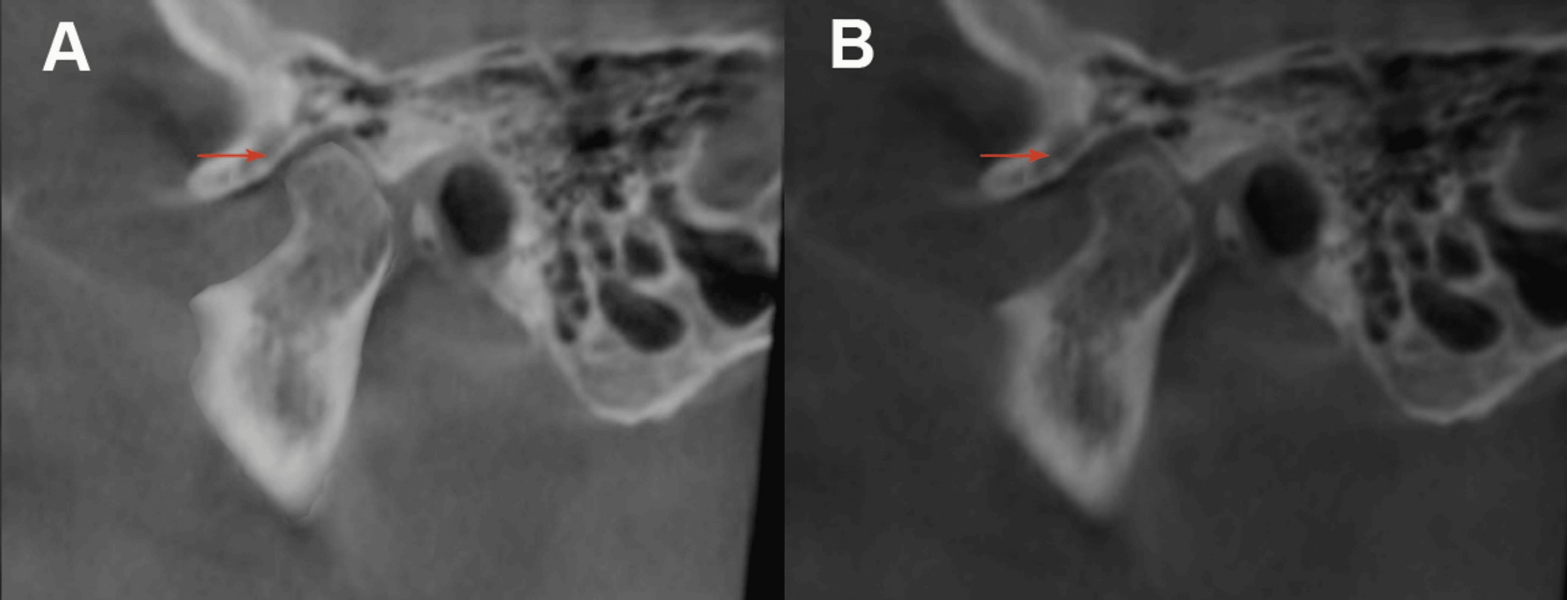
CBCT of patient showing difference in anterior space of TMJ in sagittal plane: (A) time of denture insertion; (B) after one-year interval. CBCT: cone-beam computed tomography, TMJ: temporomandibular joint.

For standardization purposes, patients with TMJ disorders were excluded from this study. Although all the patients were instructed to wear the dentures full-time except during the night, the exact hours of complete denture wear and the tolerance threshold were not considered in our study. Another confounding factor could be the varying edentulous periods in our study. To reduce operator bias, all dentures were fabricated by a single experienced prosthodontist (AHM). CBCT scans were obtained after the delivery of the complete denture, and then follow-up evaluations were performed one year after denture usage (Figure 1).

CBCT scans and clinical examinations were repeated to assess changes in TMJ space, condylar position, and interocclusal gap. Comparisons were made between post-insertion and one-year follow-up data to evaluate the impact of complete dentures on condylar dynamics.

**Figure 2:**
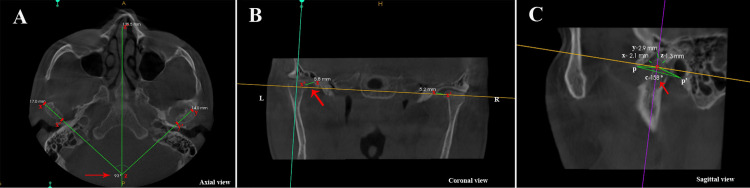
Morphometric analysis of TMJ. (A) Intercondylar angle at z point: The angle between the z-z’ mid-sagittal plane and the lines xx’ (left side) and yy’ (right side) crossing the lateral and medial prominence of the condyle in the axial view. (B) Medial space distance: xx’, the minimum distance between the medial surface of the condyle and the temporal bone in the coronal view. (C) Anterior space distance: x, the minimum distance between the anterior surface of the condyle and the temporal bone; superior space distance: y, the minimum distance between the superior surface of the condyle and the temporal bone; posterior space distance: z, the minimum distance between the posterior surface of the condyle and the temporal bone; condylar angle: pop’, where o is the center of the condyle, p is the lowermost point on the articular eminence, and p’ is the lowermost point on the mastoid process, all in the sagittal view. TMJ: temporomandibular joint.

CBCT imaging was performed using a Carestream New Generation CBCT device (Carestream Dental, Atlanta, Georgia) following a rigorous protocol (operating at a voltage of 110 kV, current of 5 mA, scan duration of 4 s, field of view (FOV) of 16 × 18 cm, resolution of 0.15 mm voxels, and slice thickness of 1 mm). To ensure uniformity in head positioning, the participants were placed in the standing position with the assistance of a head-stabilizing apparatus. Image reconstruction was performed using the CS imaging software (CS Imaging Software Version 8. Carestream Dental, Atlanta, Georgia). The intercondylar distance, condylar angle, and TMJ spaces were observed. The intercondylar angle was measured as the angle formed by a line joining the medial and lateral poles of the right and left condyles to the midsagittal plane in the axial view (Figure 2A).

The anterior, posterior, superior, and medial TMJ spaces were measured as follows: the minimum distance from the most anterior point on the condyle to the nearest point on the temporal bone was recorded as the anterior distance (x); the distance from the most posterior point on the condyle to the nearest point on the temporal bone was recorded as the posterior distance (z); the distance from the most superior point on the condyle to the nearest point on the temporal bone was recorded as the superior distance (y); and the distance from the most medial point on the condyle to the nearest point on the temporal bone was recorded as the medial distance (xx’ on the left side and yy’ on the right side), as illustrated in Figure 2B,C. The condylar angle (pop’) was measured as the angle formed between a line joining the center of the condyle (o) to the lowermost point of the mastoid process (p’) and a line joining the center of the condyle to the inferior-most point of the mastoid process (p) in the sagittal view (Figure 2C). Two blinded experts (PJ, PDT) analyzed the CBCT scans. To ensure reliability, five randomly selected CBCT scans were re-evaluated after a two-week interval. The Intraclass Correlation Coefficient (ICC) was used to assess intra- and inter-observer reliability, which was determined to be 82% for inter-observer reliability and 85% for intra-observer reliability. These results demonstrated excellent reliability and reproducibility.

Statistical analysis

The statistician (NB) was provided with coded CBCT scans to eliminate bias in data analysis. The collected measurements were systematically recorded in a Microsoft Excel spreadsheet (Microsoft Corporation, Redmond, Washington) and analyzed using IBM SPSS Statistics for Windows, Version 23 (Released 2015; IBM Corp., Armonk, New York). Descriptive statistics such as frequency distributions, means, and standard deviations were used to summarize the data. Comparisons of TMJ space and condylar morphometry between sexes were performed using an independent t-test, while paired t-tests were used to evaluate changes between denture insertion and follow-up measurements. Statistical significance was set at P < 0.05.

## Results

The mean age of the male participants was 55.43 years, while that of the female participants was 56.38 years. The difference in mean age between the sexes was not statistically significant (p = 0.484) (Table 1).

**Table 1:** Descriptive analysis of basic characteristics of study participants. p-value > 0.05 indicates non-significance; SE refers to standard error. The numbers of males and females are presented as n (%), while the age of the sample is represented as mean ± standard deviation.

Parameters	Sex	N (%)	Mean	Standard deviation	Mean SE	t-value	p-value using independent t-test
Age in years	Male	7 (46.7%)	55.42	1.98	2.41	0.78	0.484
	Female	8 (53.3%)	56.37	2.92	3.61	1.06	

In the study, no statistically significant sex-based or side-based differences were noticed for the mean difference between post-denture insertion and at one-year follow-up for all the TMJ variables except for medial space and condylar angle on the left side. The mean difference was larger in males for both variables compared to females (p<0.05), which showed that in males, as shown in Table 2.

**Table 2:** Comparison of morphometric changes in temporomandibular joint (TMJ) space between both sexes on left and right sides by independent t-test. MD: mean of difference, SD: standard deviation, TMJ: temporomandibular joint. *p-value < 0.05 is considered significant.

Parameters for TMJ space	Sex	N	Left side					Right side				
			MD between insertion and one-year follow-up	SD	95% Confidence interval of SD		p-value	MD between insertion and one-year follow-up	SD	95% Confidence interval of SD		p-value
					Upper	Lower				Upper	Lower	
Anterior space in mm	Male	7	0.40	0.22	0.29	0.08	0.692	0.40	0.21	0.28	0.05	0.308
	Female	8	0.36	0.14	0.18	0.05		0.32	0.07	0.09	0.04	
Posterior space in mm	Male	7	-0.44	0.17	0.20	0.08	0.953	-0.46	0.20	0.26	0.06	0.155
	Female	8	-0.44	0.13	0.18	0.06		-0.35	0.08	0.11	0.03	
Superior space in mm	Male	7	-0.32	0.06	0.07	0.03	0.582	-0.38	0.05	0.08	0.01	0.836
	Female	8	-0.30	0.09	0.12	0.03		-0.39	0.14	0.19	0.05	
Medial space in mm	Male	7	-0.10	0.05	0.06	0.01	0.036*	-0.10	0.03	0.04	0.01	0.448
	Female	8	-0.06	0.02	0.02	0.01		-0.12	0.03	0.04	0.01	
Condylar angle in degrees	Male	7	-4.42	0.97	1.27	0.37	0.005*	-6.71	2.62	3.30	1.15	0.764
	Female	8	-2.87	0.83	0.99	0.46		-6.37	1.59	2.07	0.74	

The analysis revealed statistically significant differences in the mean interocclusal gap and intercondylar angle at the two different time intervals. The mean interocclusal gap increased at one-year follow-up, whereas the intercondylar angle decreased (p = 0.001), as shown in Table 3.

**Table 3:** Paired t-test analysis comparing measurements at denture insertion and after one-year follow-up. *p-value < 0.05 is considered significant. SD: standard deviation, SE: standard error.

Parameters	Time interval	n	Mean	SD	SE Mean	t-value	p-value
Interocclusal gap in mm	At insertion	15	1.49	0.30	0.08	8.87	0.001*
	At one-year follow-up	15	1.79	0.39	0.10		
Intercondylar angle in degrees	At insertion	15	151.3	14.53	3.75	11.00	0.001*
	At one-year follow-up	15	148.3	15.10	3.90		

**Figure 3:**
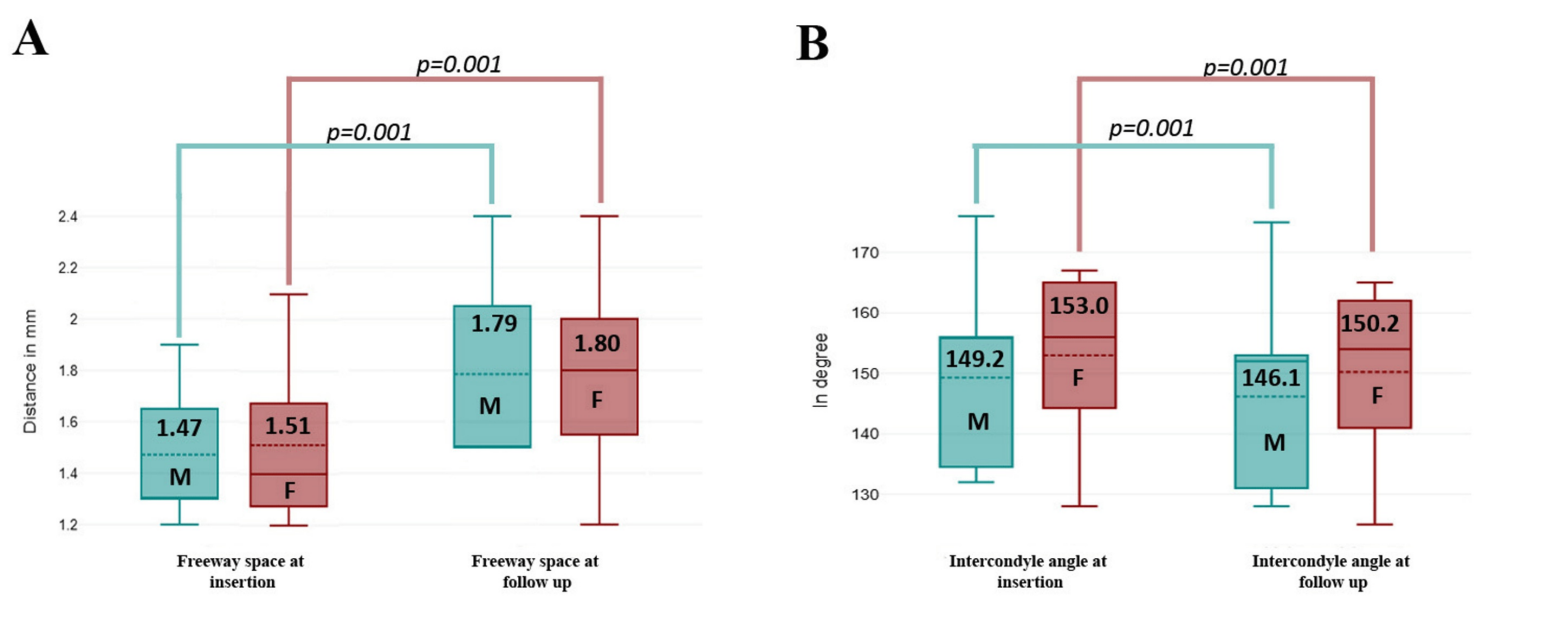
(A) Comparison of interocclusal gap (freeway space) at denture insertion and one-year follow-up between sexes, analyzed using an independent t-test. (B) Comparison of intercondylar angle at denture insertion and one-year follow-up between sexes, analyzed using an independent t-test.

The changes in the interocclusal gap and the intercondylar angle at the time of denture insertion and after a one-year follow-up, analyzed separately for males and females, revealed the following: for the interocclusal gap, the mean values were 1.47 mm for males and 1.51 mm for females at denture insertion, which significantly increased to 1.79 mm and 1.80 mm, respectively, at the one-year follow-up (p = 0.001). Similarly, for the intercondylar angle, the mean values were 149.2° for males and 153.0° for females at denture insertion, which significantly decreased to 146.1° and 150.2°, respectively, at the follow-up (p = 0.001), as shown in Figure 3.

The comparison of morphometric changes in the TMJ space between denture insertion and the one-year follow-up on both the left and right sides revealed statistically significant differences across all parameters (p = 0.001). The anterior space increased on both sides, while the posterior, superior, and medial spaces decreased, indicating that the condyle shifted posteriorly, superiorly, and medially one year after denture insertion (Table 4).

**Table 4:** Comparison of morphometric changes in TMJ space between denture insertion and after one-year follow-up on left and right side by paired t-test. *p value < 0.05 is considered significant. SE: standard error, CI: confidence interval, TMJ: temporomandibular joint.

Parameters	Left side						Right side					
	Mean of difference	SE difference	95% CI for mean of difference		t-value	p-value	Mean of difference	SE difference	95% CI for mean of difference		t-value	p-value
			Lower	Upper					Lower	Upper		
Anterior space in mm	-0.38	0.04	-0.48	-0.28	-8.45	0.001*	-0.36	0.04	-0.44	-0.27	-8.88	0.001*
Posterior space in mm	0.44	0.03	0.36	0.52	11.59	0.001*	0.36	0.04	0.27	0.45	8.67	0.001*
Superior space in mm	0.31	0.02	0.27	0.35	15.60	0.001*	0.38	0.02	0.33	0.44	14.07	0.001*
Medial space in mm	0.08	0.01	0.05	0.10	7.17	0.001*	0.11	0.00	0.09	0.13	14.45	0.001*
Condylar angle in degrees	3.60	0.30	2.94	4.25	11.78	0.001*	6.53	0.53	5.38	7.67	12.25	0.001*

Comparison of morphometric changes in the TMJ space between the left and right sides showed significant differences in the superior space (p = 0.038), medial space (p = 0.031), and condylar angle (p = 0.001). These spaces and condylar angles were greater on the right side than on the left side, indicating that the condyle on the right side was placed more superiorly and medially than on the left side (Table 5).

**Table 5:** Comparison of morphometric changes in TMJ space between right and left side at denture insertion and after one-year follow-up by paired t-test. *p-value < 0.05 is considered significant. SD: standard deviation, SE: standard error, TMJ: temporomandibular joint.

Parameters	Sides	n	Mean of difference	SD	SE mean	t-value	p-value
Anterior space in mm	Right	15	-0.36	0.16	0.04	-0.39	0.696
	Left	15	-0.38	0.18	0.05		
Posterior space in mm	Right	15	0.36	0.16	0.04	1.49	0.146
	Left	15	0.45	0.15	0.04		
Superior space in mm	Right	15	0.39	0.11	0.03	2.18	0.038*
	Left	15	0.31	0.08	0.02		
Medial space in mm	Right	15	0.12	0.03	0.01	2.27	0.031*
	Left	15	0.08	0.04	0.01		
Condylar angle in degrees	Right	15	6.53	2.07	0.53	4.77	0.001*
	Left	15	3.60	1.18	0.31		

## Discussion

This study examined variations based on sex and laterality, alongside temporal alterations in TMJ variables, interocclusal gap, and intercondylar angle subsequent to the insertion of dentures and during a one-year follow-up assessment. These results provide significant insights into the adaptations of the TMJ and the morphometric transformations correlated with denture utilization.

Age-related factors did not confound the observed results. However, the study revealed significant sex-based differences in the two TMJ parameters in the left side-medial space and condylar angle. Both variables showed larger mean differences in males compared to females. This could potentially be attributed to anatomical or biomechanical differences between the sexes, such as variations in the masticatory force or mandibular dimensions. Lewis et al. in their investigation observed variations between sexes regarding the configurations of the condylar pathways and morphologic characteristics of the articular eminence [6]. The disparities in dentoskeletal and muscular attributes observed between male and female individuals may have either led to or resulted from functional differences in mastication [7].

Notably, the study revealed statistically significant alterations in the interocclusal gap and intercondylar angle over time (p = 0.001). Both male and female subjects exhibited an increase in the interocclusal gap during the one-year follow-up, suggesting a possible modification in the vertical dimension of occlusion. The prolonged utilization of the same complete denture may lead to mandibular displacement attributed to wear of the prosthetic teeth and resorption of the residual alveolar ridge, thereby resulting in a reduction of the interocclusal space [8,9,10].

The present study reported posterior and superior placement of the condyle and reduction in posterior and superior TMJ spaces one year after complete denture insertion. This could have been due to the fact that the unilateral or bilateral diminishment of the vertical dimension of occlusion may lead to condylar retrusion [11]. The absence of posterior teeth, in particular, contributes to the posterior and superior positioning of the condyle as well as a reduction in the posterior and superior TMJ space. Even after the rehabilitation of patients with complete dentures, the masticatory muscles may not have received adequate time for reprogramming, which consequently resulted in the posterior and superior placement of the condyle at the one-year follow-up [12]. The reduction of the posterior intra-articular space could signify a constriction in the bilaminar zone, which is integral to the vascularization and sustenance of the TMJ and may also be associated with the anterior displacement of the articular disc [13].

Multiple determinants have been identified that affect the rate of acrylic tooth wear, including the patient's age and sex, as well as the occlusal force exerted by the patient [7]. Various studies have demonstrated disparate rates of wear in acrylic occlusal dental prostheses. Schmidt-Schwap et al. observed that the average vertical loss documented after one year of wearing dentures was 0.06 mm for maxillary prostheses and 0.049 mm for mandibular prostheses [14]. Consequently, to maintain vertical dimensional stability and ensure functional comfort, it is imperative that patients participate in regular follow-up appointments and consider the replacement of artificial dental materials for those experiencing significant wear if deemed necessary.

Side-based differences were also observed in this study. The superior, medial, and condylar angles were significantly greater on the right side than on the left side (p < 0.05). Condylar asymmetry may act as a marker of skeletal imbalance arising from varying growth patterns or varying remodeling influences caused by unbalanced occlusal forces [15]. Although all complete dentures were fabricated based on the concept of BA, the focus of this study was not to investigate the occurrence of unilateral chewing among the evaluated participants [16]. Future research should determine the potential relationship between condylar position asymmetry and the incidence of unilateral chewing.

Clinical implications

This study underscores the critical significance of assessing TMJ variables and interocclusal distance among individuals utilizing dentures. These results emphasize the importance of consistent follow-up consultations to identify and manage alterations in the vertical dimension, denture degradation, and TMJ adaptations. Dental professionals should inform patients regarding the potential ramifications of prolonged denture usage on mandibular alignment and advocate for the prompt replacement of deteriorated dentures to preserve occlusal stability, functional comfort, and health.

Limitations

This investigation was constrained by its observational framework, which failed to establish causal relationships. The limited sample size may impede the applicability of the results to a wider population. Furthermore, this study did not assess the potential influence of unilateral mastication, which could affect condylar asymmetry and alterations in the TMJ. A longitudinal assessment extending beyond one year is essential to comprehend the progressive adaptations of the TMJ. Additionally, variations in the techniques or materials used in the construction of dentures, which may impact wear and TMJ alterations, have not been examined. Subsequent studies should include larger and more heterogeneous samples with long-term follow-up to investigate long-term changes in the TMJ among individuals using complete dentures.

## Conclusions

Based on the findings of this study, it was concluded that sex-based differences were observed in the medial joint space and condylar angle. At the one-year follow-up visit after denture insertion, a significant decrease in the posterior, superior, and medial joint space was observed. Moreover, there was a significant increase in the interocclusal gap and a decrease in the intercondylar angle. The superior space, medial space, and condylar angles were significantly greater on the right side than on the left.
